# P210: National nosocomial infection surveillance report in Iran in 2012

**DOI:** 10.1186/2047-2994-2-S1-P210

**Published:** 2013-06-20

**Authors:** H Masoumi Asl

**Affiliations:** 1 Nosocomial Infection Department, Center for Communicable Disease Control, Tehran, Iran, Islamic Republic Of

## Introduction

Nosocomial Infection(NI) affect hundreds of millions of patients worldwide every year that lead to more serious illness, prolong hospital stays, and induce long-term disability. In 2007, a national surveillance system was established in Iran for NI based on National Nosocomial Infection Surveillance (NNIS) system definition.

## Objectives

This is the latest report of nosocomial infection surveillance during 2012 in Iran.

## Methods

In this cross sectional study four main group of NI including urinary tract(UTI), pulmonary(PNEU), surgical site(SSI) and blood stream(BSI) and other infections was investigated in 400 hospitals in 2012. Data was gathered through surveillance system that reported to center for communicable disease and analyzed in Iranian Nosocomial Infection Surveillance(INIS) software.

## Results

During the study, a total of 47380 cases have been registered. The NI rate was 0.89%. The incidence of UTI was 26.5%, PNEU 24.4%, SSI 15.5%, and BSI 15.5% respectively. The mortality rate was 13.7%. The highest incidence rate was reported in burn ward (11.8%), followed by transplantation(9.1%), ICU(7.8%), NICU(3.5%) and PICU(2.6%). 14.5% of infections were under15 years old. The most invasive measures that have been taken for cases were as follows : venous catheter (23%) , urinary catheter (18%) , suction (22%), tracheal tube(22%), ventilator(22%) and surgery(22%). 71.3% of diagnosis were laboratory based. *Ecoli*(16%), *Acinetobacter* (14%), *Pseudomonas auroginosa*(*12%*), *Klebsiella*(*11%*), *Staphylococcus aureus*(*8%*), *Entrobacter*(*7%*) *and Candida*(*6%*) were most prevalent causative agents in NI cases.

## Conclusion

The results show that it is feasible to collect data from a large number of hospitals that assist for interventions and resource allocation, but because of data under- reporting, it is necessary to encourage and change attitude of authorities and health worker by increasing commitment and holding more justification and educational sessions.

## Disclosure of interest

None declared

